# PRN Treatment of Neovascular AMD with Cycles of Three Monthly Injections

**DOI:** 10.18502/jovr.v16i2.9081

**Published:** 2021-04-29

**Authors:** Touka Banaee, Shadan Alwan, Clint Kellogg, Ilyse Kornblau, Jaafar El-Annan

**Affiliations:** ^1^University of Texas Medical Branch in Galveston, Texas, USA; ^2^University of Texas at MD Anderson, Houston, Texas, USA; ^3^Blanton Eye Institute, Houston Methodist Hospital, Houston, Texas, USA

**Keywords:** Age-related Macular Degeneration, Anti-VEGF, Naïve, Neovascular, Real World

## Abstract

**Purpose:**

To report the one and two year outcome of cycles of three, monthly anti-VEGF injections given upon reactivation of the disease in eyes with neovascular age-related macular degeneration (nAMD).

**Methods:**

Retrospective study of naïve nAMD cases with more than one year of follow-up, treated with a protocol of cycles of three monthly injections of anti-VEGF drugs upon reactivation. Visual acuity (VA) and central macular thickness (CMT) are the main outcome measures.

**Results:**

Twenty-six patients with a mean age of 78.15 ± 9.29 years (57.7% female) were included. The mean follow-up was 30.89 ± 6.95 months. Treatment started with bevacizumab in all patients but in six patients was switched to aflibercept due to inadequate response to intravitreal bevacizumab injection. The mean VA at baseline and at 12 and 24 months was 53.87 ± 21.84, 60.54 ± 21.13, and 53.68 ± 27.16 ETDRS letters, respectively. Patients gained a mean of 6.67± 13.7 (*p* = 0.013, 95% CI= 0.60 to 12.65) and 0.77±15.21 (*p* = 0.4, 95% CI: –5.65 to 7.2) letters at 12 and 24 months. CMT at baseline, 12, and 24 months was 403.55 ± 147.59, 323.95 ± 79.58, and 298.59 ± 77.161 µm, respectively. The number of injections in the first and second years were 7.65 ± 2.64 and 5.52 ± 3.01, respectively. Three eyes (12.5%) lost >15 letters at 24 months.

**Conclusion:**

This protocol can stabilize or improve vision in 87.5% of nAMD patients and can reduce the number of visits.

##  INTRODUCTION

Intravitreal injection of anti-VEGF drugs is currently the standard treatment of the neovascular form of age-related macular degeneration (nAMD). The randomized clinical trials that proved efficacy of different anti-VEGF drugs administered monthly or bimonthly intravitreal injections of these medications.^[1,2,3]^ However, fixed monthly injections protocol seems to be a great burden in a real-world setting. Less than monthly protocols have been examined; quarterly injections of ranibizumab did not meet the non-inferiority criteria compared to the monthly injections in PIER and EXCITE studies.^[4,5]^ Pro-re-nata (PRN) dosing based on pre-specified criteria of activity showed more promising results.^[6,7]^ Treat and extend (T&E) was another protocol first suggested by Spaide to lessen the burden of injections and visits.^[8]^ PRN and T&E are among the most popular ones.^[9]^

In major randomized clinical trials of anti-VEGF agents in nAMD, a loading dose of three monthly injections has usually been applied followed by fixed interval dosing protocol of injections.^[1,2,3]^ As a loading dose seems to be an accepted logic for the initial suppression of choroidal neovascular membrane (CNV) activity, it may also be more effective than just one injection in reactivations of CNV. Based on this logic, instead of giving just one injection, we gave a cycle of three successive monthly injections of anti-VEGF drugs with every reactivation of the disease (PRN protocol). This report is the visual and anatomic results at 12 and 24 months of using this protocol in the treatment of naïve nAMD eyes.

##  METHODS

This is a retrospective study of naïve eyes with nAMD treated with injection of anti-VEGF drugs in cycles of three injections upon reactivation of the disease. Data of patients who presented to one of the three retina clinics of University of Texas Medical Branch at Galveston (UTMB) from October 2014 to December 2016 and treated by a single surgeon (JE) were retrieved from the EMR of the UTMB. The study was approved by the IRB and followed the tenets of the Declaration of Helsinki. Patients who were followed for more than one year were included in this study. One eye per patient was included and in case of bilateral AMD the first involved eye was studied. There was no threshold for the level of vision for inclusion in the study.

Patients had complete ophthalmic examination including determination of best-corrected visual acuity (BCVA) with the Snellen chart, tonometry, slit lamp examination, and dilated fundoscopy at presentation and at each follow-up visit. Fluorescein angiography (FA) (HRA2, Heidelberg Engineering, Germany) was performed at presentation for all patients. Repeat FA was at the discretion of the treating physician. Optical coherence tomography (OCT) (Spectralis, Heidelberg Engineering, Germany) was performed at the initial and each follow-up visit.

Visual acuity (VA) was measured with Snellen chart, and converted to ETDRS letters based on the protocol previously described^[10]^ with the formula: approximate ETDRS letters = 85 + 50X log (Snellen fraction). LogMAR VA was calculated and used for comparison of the baseline and 12 and 24 months data. VA of counting fingers was considered equal to 1/200 or three letters of ETDRS chart.^[11]^

Anti-VEGF drugs used were aflibercept (Eylea; Regeneron, Inc., Eastview, NY), bevacizumab (Avastin; Genentech, Inc., South San Francisco, CA), and ranibizumab (Lucentis; Genentech, Inc., South San Francisco, CA). All patients were started with bevacizumab and in case of inadequate response were switched to one of the other two medications. The criteria for switching were: <100 μm reduction in central macular thickness (CMT), worsening, or no significant changes in the amount of subretinal/intraretinal fluid after three monthly injections of bevacizumab, or persistence of subretinal/intraretinal fluid after six monthly injections of bevacizumab. Injection sessions were separate from visit sessions due to the process needed for provision of the medicine.

### Protocol

At the commencement of treatment and upon discovery of disease activity at any visit, patients were treated with three monthly doses of anti-VEGF drugs. Follow-up examinations after each series of injections were scheduled monthly for the first two months; extended to bimonthly and after two bimonthly visits to every three months and continued as such if there was no activity of the disease. Patients were also instructed to check their central vision at home and return promptly in case of any new metamorphopsia, scotoma, or change in vision. Disease activity was judged based on the presence or absence of intraretinal or subretinal fluid in OCT scans, or the presence of intraretinal or subretinal blood on fundoscopy.

### Statistical Analysis

Descriptive statistics are provided as mean ±SD, median (25–75 quartiles) or percentage. VA and central foveal thickness data were analyzed with the Shapiro–Wilk test and their distribution was found not to be normal, so a comparison of vision or central foveal thickness results were made by Wilcoxon signed rank test. *P*-value < 0.05 was considered significant. Statistical analysis was performed by Statistical Package for Social Studies (SPSS) version 25 (IBM Inc., Armonk, NY).

**Figure 1:**
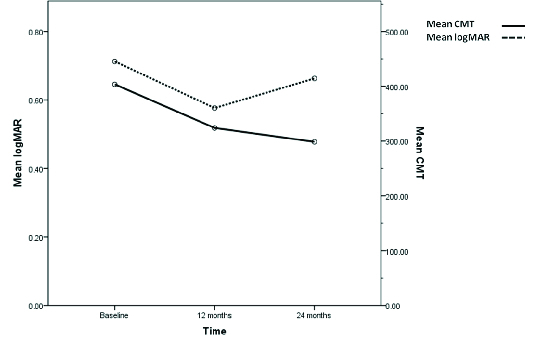
Changes in logMAR visual acuity and central macular thickness at baseline and 12 and 24 months.

##  RESULTS

Twenty six patients with a mean age of 78.15 ± 9.29 years (57.7% female) were included. The mean follow-up was 30.89 ± 6.95 months. All patients were started with bevacizumab injections (off label use), six were subsequently switched after a mean of nine injections to aflibercept. Second time switching to ranibizumab was performed in one patient. Twenty three (88.46%) patients completed two years of follow-up. One patient did not have complete follow-up of two years. One patient was lost to follow-up in the second year. Patients missed their appointments (either injection or non-injection visits) for more than one month in two cases during the first year (1.5 and 2 months of delay) and in four cases during the second year (2 ± 1 months).

VA at baseline was from 3 to 80 ETDRS letters with a mean of 53.87 ± 21.84 letters (0.65 ± 0.51 logMAR). Six (23.08%) patients had a vision of >20/40 (70 ETDRS letters) and eight (30.77%) ≤20/200 (35 ETDRS letters) at presentation.

The range of VA at 12 months was 3 to 85 with a mean of 60.54 ± 21.13 ETDRS letters (0.51 ± 0.51 logMAR) significantly different from the baseline vision (*p* = 0.013). Twelve eyes (46.15%) had a vision of >20/40 and four (15.38%) ≤20/200 at 12 months.

The range of VA at 24 months was 0 to 85 with a mean of 53.68 ± 27.16 ETDRS letters (0.68 ± 0.67 logMAR), which was not different from baseline (*p* = 0.4). Eleven (45.83%) patients had a vision of >20/40 and six (25%) ≤20/200 at 24 months.

Patients gained a mean of 6.67± 13.7 (95% CI = 0.60 to 12.65) letters at 12 months, which equals to a mean logMAR change of –0.14 ± 0.36. At 24 months, patients gained a mean of 0.77 ±15.21 (95% CI: –5.65 to 7.2) letters compared to baseline which equals to a mean change in logMAR of –0.01 ± 0.38. The number of patients with gain and loss of vision are provided in Table 1.

Changes in logMAR VA and CMT are presented in Figure 1.

Of the six patients with baseline VA of 20/40 or more (70 ETDRS letters), all had a vision of 20/40 or more at 12 months. Only one eye had a vision of <20/40 at 24 months. There were a total of 10 eyes (38.46%) at 12 months and 9 eyes (37.5%) at 24 months with a vision of 20/40 or more.

Of the eight patients with baseline VA of 20/200 or less (35 ETDRS letters), four in the first year and five in the second retained the vision of <20/200. There were a total of four (15.38%) eyes at 12 months and six (25%) eyes at 24 months with a vision of 20/200 or less. Of the six cases with a vision of 20/200 or less at 24 months, only one case had a better vision at baseline.

The mean CMT at baseline, 12, and 24 months was 403.55 ± 147.59, 323.95 ± 79.58, and 298.59 ± 77.161 µm, respectively [Figure 1]. There was a reduction of CMT relative to baseline of –88.24 ± 148.41 µm in the first year (*p* = 0.013, 95% CI = –164.81 to –14.77) and –103.16 ± 108.61 (*p* < 0.001, 95% CI = –155.50 to –50.81) µm in the second year. OCT was fluid free at 12- and 24-months encounters in 52% and 72.7% of eyes, respectively. Seven and eight eyes had intraretinal and subretinal fluid at the 12 months' visit, respectively. These numbers were 6 and 1 for the 24 months' and 6 and 3 for the last visits, respectively. Patients received a mean of 7.65 ± 2.64 injections in the first year and 5.52 ± 3.01 injections in second. The mean total number of injections over 24 months was 15 ± 6.9 given in a mean of 4.85 ± 2.18 cycles. The total number of non-injection visits was 4.92 ± 1.55 during the first year and 5.04 ± 1.46 during the second.

Seven patients (27%) achieved long-term periods of inactivity of >12 months (mean, 16.93, range, 14.5–22 months) which started in year 1 and extended into year 2. The longest injection-free period for the remaining patients was 2.95 ± 2.8 (0 to 8.00) months in the first year and 3.65 ± 2.35 (0-8.00) months in the second.

All patients were first treated with bevacizumab and six of them were switched to aflibercept after a mean of nine injections; one was further switched to ranibizumab, so we can consider them to be practically treated with bevacizumab during the first year. Of the total of seven switching episodes in six eyes, vision improved in three eyes (13.25 ± 7.68 letters), remained stable in three, and worsened in one after one cycle of treatment with the new medication, and fluid in OCT improved in five cases, and remained stable in two after one cycle of treatment.

**Table 1:** The number of patients with gain or loss of vision at 12 and 24 months


	**12 months**	**24 months**
Gain of >15 letters	12 (46.15%)	8 (33.33%)
Gain of >5 letters	13 (50%)	11 (45.83%)
<5 letters change	9 (34.61%)	7 (29.16%)
Loss of >5 letters	4 (15.38%)	6 (25%)
Loss of >15 letters	1 (4%)	3 (12.5%)

Injections were discontinued in three eyes in the second year due to the development of central geographic atrophy or futility of treatment.

##  DISCUSSION

Intravitreal injections of anti-VEGF drugs in cycles of three injections on a PRN basis in naïve neovascular AMD cases resulted in a visual gain of 6.67 letters at 12 months and a gain of 0.77 letters at 24 months. The visual results at one year are better than most real-world studies and the 24 months' results are comparable.^[12,13,14]^

Although clinical trials that proved efficacy of intravitreal injection of anti-VEGF drugs reported vision gains of 6.5–11.3 letters in the first and second years,^[1,2,3]^ real-world studies have not corroborated those results.^[12,13,14]^ In routine clinical practice as opposed to clinical trials, injections are usually given based on a variable dosing schedule rather than monthly,^[9]^ treatment is offered to all presenting with the disease and patients are not selected to fulfill certain VA, lesion size, or composition criteria. Another important difference is complying with the follow-up visits which are not as accurately followed by the patients and physicians as in a predesigned clinical trial. Many studies have explored the cause for inferior results in the real-world setting. While some have found no or weak correlations between the number of injections and visual results,^[7,14]^ others believe that there is a correlation between the number of injections and visual gain, especially when the number of injections falls <5–6 per year, the visual results get worse.^[13,15,16,17,18]^ Use of variable dosing regimens instead of monthly injections is another area of debate. Although major clinical trials such as CATT and IVAN have provided proof to the similarity of results between monthly and PRN dosing in the first year, visual results differ in favor of monthly treatment after two years (–2.4 letters difference, 95% CI, –4.8 to –0.1; *P* = 0.046, CATT trial).^[19,20,21]^ Contribution of all the aforementioned factors results in real-world treatment to be mostly preventive of further visual deterioration instead of increasing vision as expected by results of clinical trials.^[12,13,14]^ Physicians have devised newer strategies to improve the results in routine clinical practice, such as the “observe and plan” protocol.^[22,23]^ Reports of observe and plan protocol seem very promising, however, results are still not available in a real-world setting, besides the protocol seems too complicated to be followed thoroughly in a routine clinical practice.

In nearly all major RCTs of anti-VEGF agents and also in clinical practice, treatment is started with a loading dose of three monthly injections,^[3,6,24]^ which has been shown to result in better visual outcomes compared to PRN dosing from the outset.^[25]^ We speculate that for an active CNV, there is a need for continuous suppression of VEGF for at least three months until the CNV becomes quiescent and we generalized this concept to every reactivation, so we treated every reactivation of the disease with another three monthly series of injections with the hope that adoption of this strategy within an as-needed protocol may improve the results by replacing some of the non-injection visits with injection visits. This is similar to the protocol used in the IVAN study.^[20,21]^ The difference between our and IVAN's protocols is in the follow-up visits, which were kept monthly in the IVAN study but were extended upon inactivity in two successive visits in the current study, that is, incorporation of a T&E philosophy within the PRN protocol. Another major difference of the current study with IVAN is that IVAN was a prospective randomized controlled clinical trial, whereas this study is a retrospective real-world description of the results of the protocol. In spite of being a real-world study, the results of this study compare to those of IVAN [Table 2].

PRN treatment of AMD with anti-VEGF drugs was first examined in the PrONTO study, which was not a controlled study, and reported visual results comparable to the results of MARINA and ANCHOR trials. In this prospective study, patients with nAMD were treated based on visual, angiographic, and OCT objective criteria of reactivation. The study followed a strict monthly follow-up of patients and treatment was started with three monthly injections followed by an as-needed treatment. There was a +9.3 (11 median) letter improvement in the first year and 11.1 ± 12.2 letter improvement in the second. The authors of PrONTO study conclude that their results are similar to the pivotal phase III trials with lower number of injections.^[6,7]^

PRN treatment was compared with monthly regimens in some prominent RCTs. For example, in CATT study, monthly and PRN treatment of ranibizumab had a comparable visual results at 12 months. Analysis was inconclusive for bevacizumab monthly versus as needed in that trial. Worse visual results were reported at 24 months for eyes receiving PRN treatment of either ranibizumab or bevacizumab (–2.4 letters difference, 95% CI, –4.8 to –0.1; *P *= 0.046).^[19,26]^ IVAN study similarly showed that the difference in visual gain at 12 and 24 months was neither non-inferior nor inferior in PRN treatment when compared to monthly treatment.^[20,21]^

The results of T&E protocol are even more promising.^[27,28]^ In a long-term study of the T&E protocol, Berg et al^[29]^ reported a visual gain of 6.1 letters in the first year and >7.4 letters up to four years after the initiation of the treatment in a real-world setting. In a meta-analysis of real-world studies, T&E protocol had a better visual results than PRN treatment with +8.5 versus +3.5 letters improvement in the first year and +6.7 versus +1.3 letters in the second, respectively.^[30]^ Systematic reviews have also shown better results with T&E protocol compared to PRN dosing.^[27,28]^ Comparison of the PRN and T&E protocols in real-world studies have provided similar results.^[31,32,33]^

**Table:** Anatomic and visual results of selected real-world studies and clinical trials at 12 and 24 months*

**Study**	**No of patients**	**Medication used**	**Baseline**	**12 months**			**24 months**			**Notes**
		Vision ETDRS **** ***(logMAR)*** ****	CMT	Change in vision: ETDRS **** ***(logMAR)*** ****	>3 line gain in vision (%)	>3 line loss in vision (%)	Change in CMT	Mean number of injections	Change in vision: ETDRS **** ***(logMAR)*** **** *	>3 line improvement in vision (%)${}^{†}$	>3 line worsening in vision (%)${}^{†}$	Change in CMT*	Mean number of injections in two years	
Current	26	Bev/Afl/Ran	53.87 ± 21.84 **** ***(0.65 ± 0.51)*** ****	403.55 ± 147.59	6.67 ± 13.7 **** ***(*** **** **** ***0.14 ± 0.36)*** **** (*P* = 0.013)	46.15	4	–88.24 ± 148.41 *P* = 0.013	7.65 ± 2.64	0.77 ± 15.21 **** ***(0.01 ± 0.38)*** **** (*P* = 0.4)	33.33	12.5	–103.16 ± 108.61, *P* < 0.001	15 ± 6.9	Retrospective study
Rao P et al^[14]^	6723	Bev	**** ***(0.61 ± 0.57)*** ****	NA	**** ***(–0.048 ± 0.44*** **** *)*	22.7	14.3	NA	5.9 ± 2.4	NA	NA	NA	NA	NA	A real-world retrospective study of anti-VEGF monotherapy from the AAO IRIS registry
Ciulla et al^[13]^	1921	Bev (70%), Afl (13%), Ran (17%)	47.5	–			+3.1 (*p* < 0.01)		12.1	A real-world retrospective study of anti-VEGF monotherapy from the Vestrum Health Retina Database
	195	Bev (71%), Afl (12%), Ran (17%)	43.1	–	–0.7 (*p* = 0.34)		7.3			
Lotery A et al^[12]^	3350 (Ran) 4300 (Afl)	57.5 ± 21.2 (Ran) 58.5 ± 20.7 (Afl)	-0.30 ± 14.8/- (Ran) - 0.19 ± 14.7 (Afl)	10.8 (Ran) 11 (Afl)	10.6 (Ran) 10.4 (Afl)	6.70 ± 2.54 (Ran) 7.00 ± 2.40 (Afl)			Real-world study –US
IVAN study^[20,21]^	258	Discontinuous group: Bev, Ran	62.9 ± 15	4.99 ± 11.1		Median: 7 IQR: 6,9	3·5 ± 13·1		13 (8 to 17) Median (IQR)	
CATT^[19,26]^	515	As-needed groups of Bev, Ran	60.4 ± 13.4 (Bev) 61.5 ± 13.2 (Ran)	461 ± 175 (Bev) 458 ± 193 (Ran)	+5.9 ± 1.0 (Bev) +6.8 ± 0.8 (Ran)	28 (Bev) 25 (Ran)	8 (Bev) 5 (Ran)	-152 ± 11 (Bev) -168 ± 11 (Ran)	7.7 ± 3.5 (Bev) 6.9 ± 3.0 (Ran)	5·87 ± 16·3	28.3 (Bev) 30.7 (Ran)	11.6 (Bev) 7.2 (Ran)	–153 ± 189 (Bev) –166 ± 190 (Ran)	14.1 ± 7.0 (Bev) 12.6 ± 6.6 (Ran)	
PrONTO^[6,7]^	40	PRN, Ran	56.2 median: 57	393.9 median: 384.5	+9.3 median: +11.0	35	5	–177.8 median: -185.5	5.6 ± 2.3	11.1 ± 12.2	43	2.5	–212	9.9 ± 5.3	No correlations between baseline vision or lesion size and the number of injections. No correlations between visual outcomes and the number of injections
Observe and plan^[22]^	102 (112 eyes)	Afl	61.8 ± 15.4	438 ± 148	8.0 ± 12.0	26%	1%	–154	8.7 ± 3.0	6.2 ± 14.6	20%	8%	–150	15.3 ± 5.2	Prospective
Observe and plan^[36]^	104 (115 eyes)	Ran	58.3 ± 18	342 ± 85	9.7	30	3	–99	7.8 ± 3.1	9.2	33	4	–96	13.6 ± 0.1	Prospective
*All numbers are in the form of Mean ± SD unless stated otherwise; ${}^{†}$Changes compared to baseline. AAO, American Academy of Ophthalmology; Afl, aflibercept; Bev, bevacizumab; CATT, comparison of AMD treatment trial; CMT, central macular thickness; IQR, interquartile range; IRIS registry, Intelligent Research in Sight registry; IVAN, inhibit VEGF in age-related choroidal neovascularization trial; PrONTO, prospective OCT imaging of patients with neovascular AMD treated with intraocular ranibizumab; Ran, Ranibizumab															

In the routine clinical practice, physicians mostly use the PRN or T&E protocols for the treatment of nAMD,^[9]^ so the real-world studies reported based on electronic databases usually include patients treated by these two protocols in practice. In spite of the promising results of clinical trials, and aforementioned studies, visual results of real-world studies are not very impressive. Rao et al^[14]^ in a real-world study of intravitreal injection of anti-VEGF drugs for the treatment of AMD in the US, based on data from the Intelligent Research in Sight (IRIS) registry, compared the one-year outcomes of the injections of bevacizumab, ranibizumab, and aflibercept. Patients gained –0.04 to –0.053 logMAR in the first 12 months with a mean of 5.9–6.4 injections of bevacizumab, ranibizumab, or aflibercept. Similarly, Ciulla et al^[13]^ reporting results from Vestrum Health Retina Database from the US stated that there was no change of vision (–0.7 ETDRS letters, *p* = 0.43) at 12 months with 7.3 injections of the three drugs. Lotery et al^[12]^ found a change of -0.30 ± 14.8 and -0.19 ± 14.7 letters with 6.7–7 injections of ranibizumab and aflibercept, respectively, at 12 months in cases retrieved form a standard EMR in the US.

Although different studies cannot be directly compared with each other, with a mean gain of 6.67 ± 13.7 ETDRS letters at 12 months and 0.77 ± 15.21 at 24 months, results of the current study seem to be better than the results of most real-world studies in the US and compares to the results of the IVAN trial, which was a prospective study [Table 2]. Having similar results at 12 and 24 months to a prospective study is promising. As irregular follow-ups can be a cause of poor visual outcomes in the real-world studies, having few missed appointments in this study may discuss the similarity of results with a prospective study. However, in the current study, the follow-ups were extended to three months, mostly in the second year, while in the IVAN study, monthly follow-ups were pursued through the second year. Undertreatment has been stated as an important factor causing lower visual results in real-world settings.^[13]^ The number of injections in the first and second years in the current study was 7.65 ± 2.64 and 5.52 ± 3.01, respectively, for a cumulative number of 15 ± 6.9 during the two years. This number compares with both the real-world studies and the PRN groups of IVAN, CATT, and PrONTO studies [Table 2].

The number of non-injection visits in the first and second years was 4.92 ± 1.55 and 5.04 ± 1.46, respectively. The current study was performed where bevacizumab would usually be injected in a separate visit because of logistics in preparation of the medication. If same-day injection was possible, the number of non-injection visits would certainly be lower than the current results. We also believe that with the aforementioned situation, this protocol of PRN cycles of injections has saved patients many unnecessary visits. Also, the PRN nature of decision-making has spared patients from many injections. Indeed, 27% of patients in this study achieved inactivity of the disease for 14–22 months without injections.

In the current study, OCT was fluid-free in 52% and 72.7% at 12 and 24 months, respectively. The corresponding rates in the IVAN study were 31 and 37% in the discontinuous treatment group.^[20,21]^ In CATT study, 19 and 13.9% of eyes in the PRN bevacizumab group and 23.9 and 22.3% of eyes in the PRN ranibizumab group were without any fluid in OCT at 12 and 24 months, respectively.^[19,26]^ The monthly treatment groups of both IVAN and CATT had higher rates of absence of fluid in OCT at 12 and 24 months: 44 and 54% for the continuous treatment group – IVAN study; 26 and 30.2% in the bevacizumab monthly – CATT study; and 43.7 and 45.5% for ranibizumab monthly – CATT study. In a study by Hatz et al,^[32]^ which was a prospective study comparing the T&E and PRN protocols, the rate of absence of any fluid in OCT at 12 months was 67% in the T&E and 47% in the PRN group. The results of our study seem to be comparable to the results of this study, sitting somewhere between the PRN and T&E groups.

There is a worse visual outcome in the second year in spite of less activity of the disease in OCT [Figure 1]. As we did not restrict inclusion of patients based on VA levels, lesion size, or components, it is possible that the development of atrophy or scar in eyes with more advanced lesions during the second year of the study have skewed the results toward lower vision range.

This study is a retrospective study and limitations of retrospective studies apply to it. One of the major limitations is that VA is not measured by the ETDRS chart and is reported as approximate ETDRS letters which is a calculated number. Some real-world studies have provided that patients who are lost to follow-up have worse vision at their last visit compared to those who are followed-up.^[13,17,34,35]^ Thus, by not including patients lost to follow-up before one year, we may have introduced a selection bias toward patients with better visual results. The small number of patients is another limitation of the current study. As previously stated, because of the unavailability of same-day injections, the number of non-injection visits may have been overestimated.

In conclusion, the visual results of the current study in the first year are very promising. The protocol is easy to implement and the compliance rate is very high. In the second year of the study, there is a decrease in the gained letters of VA in spite of a higher rate of inactivity of the lesions in OCT. Continuing monthly follow-ups may reduce the vision loss in the second year.
